# Editorial: Protein-Material interfaces: Fundamentals and applications volume II

**DOI:** 10.3389/fmolb.2023.1137613

**Published:** 2023-01-17

**Authors:** Greta Faccio, Prashanth Asuri

**Affiliations:** ^1^ Independent Researcher, St. Gallen, Switzerland; ^2^ Department of Bioengineering, Santa Clara University, Santa Clara, CA, United States

**Keywords:** functional surface, protein immobilisation, functional material, biosensor, enzyme immobilization

When scientists work at the interface and investigate how to tailor it, they can unlock a plethora of possibilities for the future of different sectors. This is well exemplified by the five articles published as a part of this research topic; these works can contribute to development of natural delivery systems for cosmeceutical applications, bio-inspired functional surfaces for biomedical, biosensing, and food packaging, as well as techniques for the measurement of protein adsorption. Following from the previous research topic (Editorial: *Protein-Material interfaces: Fundamentals and applications*), this second volume explores how proteins can interact with surfaces and how these interactions may be intentionally applied to alter surface properties and introduce novel functionalities.

We can understand and study only what we can measure and the review article on nanomechanical mass spectrometry presents the crucial role of nanomechanical techniques in the understanding of protein deposition at the surface. This research topic also features a review article on hydrophobins, a protein with a tight affinity for surfaces that can self-assemble at hydrophobic/hydrophilic interfaces into amphipathic layers, and shines light on the potential applications of hydrophobins from fungi.

As a complementary approach to lab studies, *in silico* predictions of protein structures often offers access to information that is inaccessible experimentally through X-ray crystallography or electron microscopy. Using a protein structure prediction program (AlphaFold2, AF2), the conformation of the adhesion devices of *Streptococcus thermophilus* was studied. This study demonstrates the ability of AF2 to unveil structural and functional details of the molecular machineries involved in adhesion of bacteriophages to their hosts.

The important issue of sustainability has also received attention in the submitted works; alginate and chitosan polymers has been investigated by two papers – one that describes the delivery of bee products for cosmeceuticals and medical applications, and a second that explores nanocomposite film based-sensors based on catalase.

What will the future bring? Hopefully a better understanding of the single molecule events at the surface and ways to better tune and exploit them, while the current innovations reach the market and deliver impact.

